# Timing of Flap Coverage in Open Fractures: A Systematic Review and Meta-Analysis

**DOI:** 10.7759/cureus.107338

**Published:** 2026-04-19

**Authors:** Sina Dehnadi, Rocio Pérez, Ming Zien Yu, Nader Ameri, Senthooran Kathiravelupillai, Panagiotis Bompolas, Ishith Seth

**Affiliations:** 1 Accident and Emergency, Royal Free Hospital NHS Foundation Trust, London, GBR; 2 Plastic Surgery, Policía Nacional del Perú, Lima, PER; 3 Plastic Surgery, School of Medical Sciences, Universiti Sains Malaysia, Jalan Universiti, Pinang, MYS; 4 Orthopedic Surgery, Istituto Ortopedico Rizzoli, Bologna, ITA; 5 Accident and Emergency, Kingston and Richmond NHS Foundation Trust, London, GBR; 6 Trauma and Orthopedics, Buckinghamshire Healthcare NHS Trust, Aylesbury, GBR; 7 Plastic Surgery, St. Vincent's Medical Center, Melbourne, AUS

**Keywords:** flap-based reconstruction, lower limb trauma, open fracture wounds, orthoplastic, systematic review and meta-analysis, timing of the surgery

## Abstract

Open tibial fractures remain the most prevalent open fracture, necessitating orthoplastic management with flap reconstruction. Early soft tissue coverage, ideally within 72 hours, is widely regarded as the gold standard for reducing infection, promoting bone healing, and improving limb salvage. However, injury severity, patient comorbidities, and pre-theater coordination pose challenges to the recommended management window, thereby increasing the number of patient-reported complications. A systematic review and meta-analysis were conducted in accordance with the Preferred Reporting Items for Systematic Reviews and Meta-Analyses (PRISMA) guidelines and were prospectively registered with PROSPERO (CRD420251033412). A comprehensive search of six databases identified comparative studies evaluating flap reconstruction performed within 72 hours versus beyond 72 hours following injury. The primary outcome addressed operative complications. Risk of bias was assessed using ROBINS-I (Cochrane Collaboration, London, UK) and RoB2 (Cochrane Collaboration, London, UK), while evidence quality and methodology review were evaluated using GRADE and AMSTAR-2. From 2010 to 2025, 14 articles met the inclusion criteria among 21 potentially eligible studies, comprising 2652 patients (1859 males and 793 females). Early reconstruction within 72 hours was associated with fewer complications compared with delayed reconstruction. Meta-analysis demonstrated lower risks of infection, osteomyelitis, and amputation (risk ratios of 0.69, 0.28, and 0.55; confidence intervals of 0.47-1.02, 0.16-0.49, and 0.27-1.12, respectively), with a statistically significant reduction in osteomyelitis. Prior reviews had critical methodological flaws, whereas this study achieved a high confidence rating. Flap coverage within 72 hours is associated with improved clinical outcomes and should be prioritized whenever feasible for patients with open fractures of their lower limbs. Despite guideline recommendations, the majority of flap reconstructions are still performed more than 72 hours after injury. This highlights the need for improved multidisciplinary coordination and timely access to specialist orthoplastic care.

## Introduction and background

The incidence of open fractures is increasing, with open tibial fractures being the most common [1]. These represent some of the most complex and high-risk injuries, involving soft tissue damage, bone exposure, and a high incidence of complications. Suboptimal management increases the risk of complications, including infections and nonunions, necessitating reoperations [2]. Optimizing evidence-based soft tissue reconstruction practice is therefore critical to improving outcomes.

The management of open fractures, according to the Arbeitsgemeinschaft für Osteosynthesefragen (AO) Foundation’s Principles, emphasizes that early wound coverage is crucial for reducing infection and accelerating healing, and that the primary factor influencing outcomes is the extent of soft tissue injury [3,4]. This approach is often delivered through orthoplastic management, collaborating orthopedic and plastic surgical specialties to optimize skeletal stabilization and soft tissue reconstruction. Godina’s 1986 study established the foundation for modern timing principles in reconstructive microsurgery. In a 532-patient cohort, he showed that flap coverage, the transfer of vascularized tissues to cover soft tissue defects and exposures, performed within 72 hours of admission, was associated with significantly improved outcomes, reduced susceptibility to flap failure and postoperative infection rates, improved bone healing, and shorter hospitalization stays compared with delayed reconstruction [5]. These findings are consistent with current clinical consensus and the results of systematic comparative reviews, establishing early soft tissue reconstruction as the gold standard in modern extremity trauma care.

Historically, delayed closure, occurring four to six days after the injury, remained common practice. Although early studies, including Daniel et al., demonstrated improved outcomes with early microvascular flap coverage, adoption was inconsistent, and optimal timing remained uncertain [6]. Wood et al. subsequently conducted the first systematic review and meta-analysis (SRMA) on flap coverage timing, reporting higher infection rates with delays beyond 72 hours and showing variations related to host comorbidities, fracture severity, and wound contamination [7]. Despite these advances, practice variations persist globally, highlighting the need to define optimal timing.

The objective of this study was to conduct a methodologically robust SRMA to evaluate the impact of early versus delayed soft tissue reconstruction on clinical outcomes following lower-limb fractures. Additionally, the quality of available evidence was reviewed to generate evidence-based recommendations to guide and support clinical decision-making and optimize patient outcomes.

## Review

Methodology

**Figure 1:**
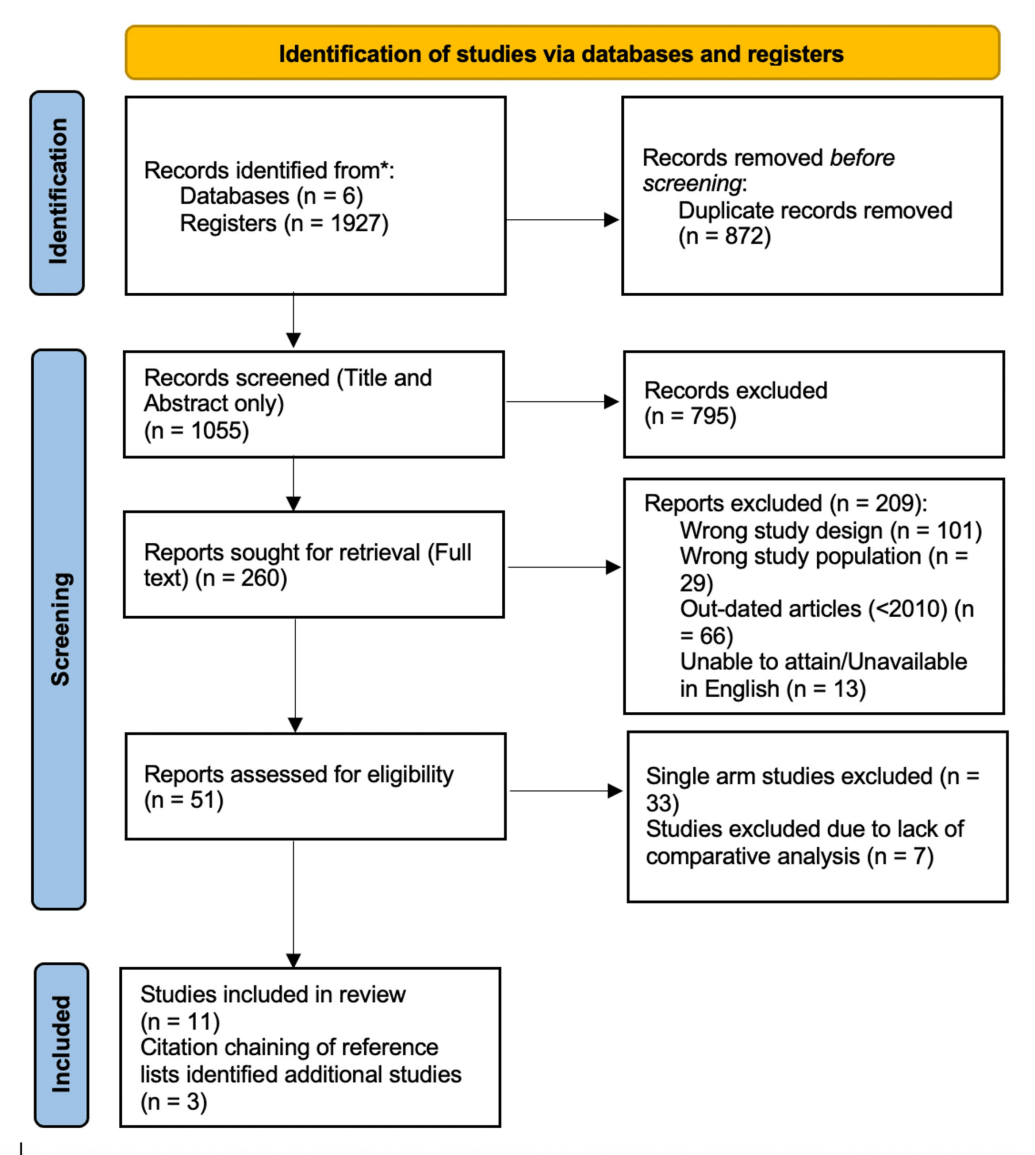
PRISMA flowchart PRISMA: Preferred Reporting Items for Systematic Reviews and Meta-Analyses

This SRMA was conducted in accordance with the Cochrane Handbook for Systematic Reviews of Interventions [8]. The protocol was prospectively registered on PROSPERO (CRD420251033412) and reported using the Preferred Reporting Items for Systematic Reviews and Meta-Analyses (PRISMA) 2020 flow diagram software [9]. The selection of articles is documented in the PRISMA flow diagram, with reasons for exclusion provided at each stage (Figure 1).

Search Strategy

A literature search was conducted in PubMed, the Cochrane Library, Embase, Web of Science, MEDLINE, and Scopus from 2010 to July 2025. The year 2010 was selected to ensure the inclusion of studies that reflect contemporary orthoplastic practice. Eligibility was limited to full-text, peer-reviewed studies published in English that involved human subjects and used a comparative study design. These designs include randomized and non-randomized controlled trials (non-RCTs), cohort studies, and case-control studies. A search was then adapted for the aforementioned databases.

Study Eligibility

Studies were eligible for inclusion if they involved human participants with open fractures of the lower limb (e.g., Gustilo-Anderson types II, IIIA, IIIB, and IIIC) aged over 16 years and directly compared early flap coverage (≤72 hours) with delayed flap coverage (>72 hours). Only comparative study designs were included, comprising randomized control trials (RCTs), prospective/retrospective cohort studies, and case-control series. Additionally, only studies published from 2010 onwards were considered.

Studies were excluded if they were editorials, expert opinions, narrative reviews, or systematic reviews, or if they focused on closed fractures, chronic wounds, pressure ulcers, or non-traumatic defects. Animal or cadaveric studies were also excluded, as were studies that did not clearly specify the timing of flap coverage or did not compare early versus delayed reconstruction. Furthermore, articles not published in English were excluded from the analysis.

Study Selection and Data Extraction

The extracted articles were exported to Rayyan, and duplicates were removed. Two reviewers (S.D. and M.Z.Y.) independently screened the articles, titles, and abstracts. Eligibility discrepancies were then resolved by a third independent reviewer (R.P.H.). Full-text eligibility reviews were then conducted independently by the aforementioned reviewers. A standardized data extraction screening form in Google Sheets (Google, Mountain View, CA, USA) was used.

A predesigned data extraction sheet was used to collect all variables needed for this review; three independent reviewers extracted relevant data and resolved the discrepancies by consensus. The extracted data included the study's first author, year of study, country, number of participants, flap coverage times, flap types, complications (e.g., infections, flap complications, nonunion, amputations, reoperations), funding status, and conflicts of interest. The patient characteristics are age, sex, and fracture type. The intervention characteristics were flap coverage type and time since injury.

Risk of Bias and Quality Assessment

The risk-of-bias assessment was conducted using only the Risk of Bias in Non-Randomized Studies of Interventions (ROBIN-I; Cochrane Collaboration, London, UK) tool due to the lack of randomized clinical trials [10]. Publication bias was not formally assessed due to the limited number of included studies. In all comparative studies, the quality of evidence was assessed using the Grading of Recommendations, Assessment, Development, and Evaluation (GRADE) tool [11].

Data Analysis

The statistical analyses of the data were performed in R version 4.5.2 (R Foundation for Statistical Computing, Vienna, Austria) using the mada, meta, and metafor packages. Dichotomous outcomes were pooled as risk ratios with 95% confidence intervals, and statistical heterogeneity was assessed using the I² statistic, τ², and Cochran’s Q test. A p-value < 0.05 was considered statistically significant.

Meta-Analysis

A meta-analysis was performed on four major complications: infections, amputations, osteomyelitis, and reoperations. A random-effects Mantel-Haenszel model was used, with a continuity correction of 0.5 applied to studies with zero cells; this approach was considered appropriate given the low event rates and the need to retain clinically relevant data. The random-effects model was used to account for heterogeneity across studies.

Results

An initial database search yielded 1927 studies, of which 18 were two-arm comparative studies evaluating flap coverage ≤72 hours versus >72 hours. Eleven studies reported comparative complication outcomes stratified by timing and were therefore included in the complication analysis. A thorough review of reference links for similar SRMA studies identified an additional three articles for consideration, bringing the total to 14 comparative studies (Table 1). Of these, 10 were conducted retrospectively and 4 prospectively, with varying levels of evidence: II (n = 3), III (n = 7), and IV (n = 4).

**Table 1:** Study characteristics N/A: not applicable

Study ID	Study title	Author	Country	Study design	Patient number	Mean age	Fracture type (Gustilo-Anderson classification)	Flap coverage <72 hours (intervention group) - patient number	Flap coverage >72 hours (control group) - patient number	Flap coverage type	Infections	Osteomyelitis	Reoperations	Amputations	Thrombosis	Nonunion	Ulcer	Flap failure/necrosis/complications	Funding	Conflict of interest
1	Outcomes of early fixation and soft tissue coverage in type IIIB open proximal tibia fractures: a prospective study [12]	Poonia et al.	India	Prospective cohort study	Total: 56, males: 43, females: 13	38.5	Type IIIB	28	28	Free flap: 22, local flap: 34	<72 hours: 5, >72 hours: 9	N/A	<72 hours: 4, >72 hours: 8	N/A	N/A	<72 hours: 3, >72 hours: 7	N/A	<72 hours: 1, >72 hours: 4	N/A	None
2	The continued impact of Godina’s principles: outcomes of flap coverage as a function of time after definitive fixation of open lower extremity fractures [13]	Le et al.	USA	Retrospective cohort study	Total: 863, males: 586, females: 277	45.7	Mixed (II and III)	145	718	N/A	<72 hours: 24, >72 hours: 187	<72 hours: 8, >72 hours: 164	<72 hours: 64, >72 hours: 660	N/A	N/A	N/A	N/A	N/A	None	N/A
3	The effect of free versus local flaps on time to union in open tibia fractures [14]	Zelenski et al.	USA	Retrospective cohort study	Total: 118, males: 39, females: 79	41	Mixed (14/118 IIIA, 96/118 IIIB, 7/118 IIIC)	24	94	Free flap: 66, local flap: 52	N/A	N/A	N/A	N/A	N/A	<72 hours: higher rate of nonunion, >72 hours: lower rate of nonunion	N/A	N/A	N/A	None
4	What is the safe window from definitive fixation to flap coverage in type 3B open tibia fractures? Supporting plastics and orthopaedics alliance in reducing trauma adverse events (SPARTA) [15]	Al-Hourani et al.	UK	Retrospective cohort study	Total: 373, males: 267, females: 106	42.4	Type IIIB	235	138	Free flap: 193, local flap: 180	<72 hours: 35, >72 hours: 32	N/A	N/A	N/A	N/A	N/A	N/A	N/A	N/A	None
5	Delay in flap coverage past 7 days increases complications for open tibia fractures: a cohort study of 140 North American trauma centers [16]	Pincus et al.	Canada	Retrospective cohort study	Total: 672, males: 488, females: 184	42	Mixed (IIIB and IIIC)	130	542	N/A	<72 hours: 3, >72 hours: 49	<72 hours: -, >72 hours: 24	N/A	<72 hours: 7, >72 hours: 53	<72 hours: 8, >72 hours: 40	N/A	<72 hours: 1, >72 hours: 35	N/A	N/A	None
6	The lymphatic response to injury with soft-tissue reconstruction in high-energy open tibial fractures of the lower extremity [17]	Van Zanten et al.	Australia	Prospective cohort study	Total: 17, males: 15, females: 2	44	Type IIIB	3	14	Free flap: 9, local flap: 7, skin graft: 1	<72 hours: 1, >72 hours: 3	<72 hours: 1, >72 hours: 6	N/A	N/A	N/A	N/A	N/A	N/A	None	N/A
7	Treatment of compound tibial fracture with free osteomuscular latissimus dorsi scapula flap [18]	Junnila et al.	Finland	Prospective cohort study	Total: 19, males: 18, females: 1	42.3	Mixed (2/19 IIIA, 15/19 IIIB, 2/19 IIIC)	1	18	Free flap: 19, local flap: -	<72 hours: -, >72 hours: 2	N/A	N/A	N/A	<72 hours: 1, >72 hours: 2	<72 hours: -, >72 hours: 4	N/A	N/A	N/A	N/A
8	Early versus late flap coverage for open tibial fractures [19]	Chua et al.	Singapore	Retrospective cohort study	Total: 89, males: 83, females: 6	38.3	Mixed (81/89 IIIB and 8/89 IIIC)	30	59	Free flap: 39, local flap: 50	<72 hours: 8, >72 hours: 37	N/A	N/A	<72 hours: -, >72 hours: 4	N/A	N/A	N/A	<72 hours: 6, >72 hours: 5	N/A	None
9	Gustilo IIIB open tibial fractures: an analysis of infection and nonunion rates [20]	Singh et al.	Singapore	Retrospective cohort study	Total: 103, males: 111, females: 8	38.2	Type IIIB	59	44	N/A	<72 hours: 15, >72 hours: 21	N/A	N/A	N/A	N/A	N/A	N/A	N/A	None	None
10	Complications and timing of soft tissue coverage after complete articular, open tibial plateau fractures [21]	Grisdela et al.	Denmark	Retrospective cohort study	Total: 50, males: 36, females: 14	45.7	Mixed (8/50 II, 24/50 IIIA, 13/50 IIIB, 5/50 IIIC)	23	27	Free flap: 34, local flap: 12, skin graft: 5	<72 hours: 7, >72 hours: 13	N/A	<72 hours: 23, >72 hours: 14	<72 hours: 1, >72 hours: 1	N/A	<72 hours: 1, >72 hours: 3	N/A	N/A	None	None
11	Single-stage orthoplastic reconstruction of Gustilo-Anderson grade III open tibial fractures greatly reduces infection rates [22]	Mathews et al.	UK	Retrospective cohort study	Total: 73, males: 25, females: 48	46.25	Mixed (5/74 IIIA, 66/74 IIIB, 3/74 IIIC)	25	48	Free flap: 41, local flap: 18, skin graft: 3, direct closure: 11	<72 hours: 5, >72 hours: 6	N/A	N/A	N/A	N/A	N/A	N/A	N/A	N/A	None
12	Markers of blood coagulation and fibrinolysis in patients with early and delayed microsurgical reconstructions in the lower extremities [23]	Kloeters et al.	UK	Prospective cohort study	Total: 70, males: 40, females: 30	40.5	N/A	35	35	Free flap: 52, local flap: 28	N/A	N/A	<72 hours: 2, >72 hours: 5	N/A	<72 hours: 2, >72 hours: 5	N/A	N/A	N/A	Yes	None
13	Early soft tissue coverage and negative pressure wound therapy optimises patient outcomes in lower limb trauma [24]	Liu et al.	Australia	Retrospective cohort study	Total: 103, males: 90, females: 13	42.3	Mixed (6/103 IIIA, 78/103 IIIB, 19/103 IIIC)	24	81	N/A	<72 hours: 1, >72 hours: 19	<72 hours: 1, >72 hours: 12	N/A	N/A	N/A	<72 hours: 4, >72 hours: 21	N/A	<72 hours: 3, >72 hours: 12	N/A	None
14	When is the critical time for soft tissue reconstruction of open tibia fracture patients? [25]	Lee et al.	Korea	Retrospective cohort study	Total: 46, males: 33, females: 13	41.5	Mixed (IIIB and IIIC)	10	36	N/A	N/A	<72 hours: 1, >72 hours: 9	<72 hours: 1, >72 hours: 3	N/A	N/A	N/A	N/A	<72 hours: 1, >72 hours: 15	Yes	N/A

Across all 14 studies, a total of 2652 patients were analyzed, most of whom had Gustilo-Anderson IIIB fractures of the lower limb. Among the 10 studies reporting fracture severity, there were 8 type II, 51 type IIIA, 898 type IIIB, and 44 type IIIC injuries. Four studies (study IDs 2, 5, 12, and 14) did not specify the Gustilo-Anderson classification. The gender distribution favored males, with a male-to-female ratio of 2.24:1 (1763 male patients and 786 female patients), and a mean age of 41.8 years. One study (study ID 9) did not clearly specify the gender of the patients included.

Of the 2652 patients within the complication analysis, 772 (29.1%) flap coverages were received within 72 hours, and 1882 (70.9%) flap coverages were received beyond 72 hours. Across these groups, a range of complications occurred; however, the number and percentage of complications were higher in the delayed-coverage group. The most common complications were infections, comprising superficial and deep infections and osteomyelitis, with incidence rates increasing from 14.94% to 22.02% and from 3.53% to 15.46%, respectively. Reoperations increased from 30.29% to 81.75%. This numerical imbalance between early and delayed flap groups should be acknowledged as a potential source of weighting bias and is further addressed in the limitations of this review. One study (study ID 5) reported an eightfold increase in ulceration with delayed coverage, which was the most significant change. Other complications, including nonunion, amputation, thrombosis, and flap-related complications, also had a higher incidence when flap coverage exceeded the recommended 72-hour limit. A separate analysis (study ID 1) examined differences in antibiotic requirements beyond four weeks, time to fracture union, and hospital stay duration, all of which demonstrated more favorable outcomes with early flap coverage. Antibiotic use beyond four weeks was halved, fracture union time occurred more than five weeks earlier, and hospital stays were shortened (six days) in the <72-hour group.

Risk of Bias Assessment

The 14 studies were assessed for risk of bias using ROBINS-I (Table 2). According to this evaluation, all studies showed a serious risk of bias, with two studies demonstrating a critical risk (study IDs 9 and 11). Formal assessment of publication was not performed due to the limited number of included studies. However, publication bias cannot be ruled out, particularly given the tendency for studies with positive findings to be preferentially reported.

**Table 2:** ROBIN-I

Study ID	Title	Author	Domain 1	Domain 2	Domain 3	Domain 4	Domain 5	Domain 6	Domain 7	Overall
1	Outcomes of early fixation and soft tissue coverage in type IIIB open proximal tibia fractures: a prospective study [12]	Poonia et al.	Low	Low	Low	Low	Low	Serious	Low	Serious
2	The continued impact of Godina’s principles: outcomes of flap coverage as a function of time after definitive fixation of open lower extremity fractures [13]	Le et al.	Serious	Low	Moderate	Low	Low	Low	Low	Serious
3	The effect of free versus local flaps on time to union in open tibia fractures [14]	Zelenski et al.	Serious	Low	Moderate	Low	Low	Low	Low	Serious
4	What is the safe window from definitive fixation to flap coverage in type 3B open tibia fractures? Supporting plastics and orthopaedics Alliance in reducing trauma adverse events (SPARTA) [15]	Al-Houraniet al.	Serious	Low	Moderate	Low	Low	Low	Low	Serious
5	Delay in flap coverage past 7 days increases complications for open tibia fractures: a cohort study of 140 North American trauma centers [16]	Pincus et al.	Serious	Low	Low	Low	Low	Low	Low	Serious
6	The lymphatic response to injury with soft-tissue reconstruction in high-energy open tibial fractures of the lower extremity [17]	van Zanten et al.	Serious	Low	Low	Serious	Serious	Low	Moderate	Serious
7	Treatment of compound tibial fracture with free osteomuscular latissimus dorsi scapula flap [18]	Junnila et al.	Serious	Low	Low	Serious	Serious	Low	Serious	Serious
8	Early versus late flap coverage for open tibial fractures [19]	Chua et al.	Serious	Low	Low	Low	Low	Low	Moderate	Serious
9	Gustilo IIIB open tibial fractures: an analysis of infection and nonunion rates [20]	Singh et al.	Critical	Low	Low	Low	Critical	Low	Moderate	Critical
10	Complications and timing of soft tissue coverage after complete articular, open tibial plateau fractures [21]	Grisdela et al.	Serious	Low	Low	Serious	Serious	Low	Moderate	Serious
11	Single-stage orthoplastic reconstruction of Gustilo-Anderson grade III open tibial fractures greatly reduces infection rates [22]	Mathews et al.	Serious	Low	Low	Serious	Critical	Moderate	Moderate	Critical
12	Markers of blood coagulation and fibrinolysis in patients with early and delayed microsurgical reconstructions in the lower extremities [23]	Kloeters et al.	Serious	Low	Moderate	Low	Serious	Low	Moderate	Serious
13	Early soft tissue coverage and negative pressure wound therapy optimises patient outcomes in lower limb trauma [24]	Liu et al.	Serious	Low	Moderate	Low	Low	Low	Moderate	Serious
14	When is the critical time for soft tissue reconstruction of open tibia fracture patients? [25]	Lee et al.	Serious	Low	Moderate	Low	Low	Low	Moderate	Serious

Methodological Quality Assessment

The included studies were assessed for methodological quality using the GRADE approach, with most rated as of moderate quality. They all shared a concern about the lack of blinding, largely because the studies were conducted retrospectively. Imprecision due to small sample sizes was also noted in a few studies. Ultimately, the risk of bias ranged from moderate to high (Table 3).

**Table 3:** Methodology quality assessment

Study ID	Title	Author	Year	Study design	Allocation of concealment	Lack of blinding/bias	Selective reporting of outcomes	Inconsistency	Imprecision	Indirectness/publication bias	Risk of bias	Quality
1	Outcomes of early fixation and soft tissue coverage in type IIIB open proximal tibia fractures: a prospective study [12]	Poonia et al.	2024	Prospective cohort study	Moderate risk	High risk	Low risk	Low risk	Moderate risk	Low risk	Moderate risk	Moderate quality
2	The continued impact of Godina’s principles: outcomes of flap coverage as a function of time after definitive fixation of open lower extremity fractures [13]	Le et al.	2024	Retrospective cohort study	N/A	High risk	Moderate risk	Low risk	Low risk	Low risk	Moderate risk	Moderate quality
3	The effect of free versus local flaps on time to union in open tibia fractures [14]	Zelenski et al.	2021	Retrospective cohort study	N/A	High risk	Moderate risk	Low risk	Low risk	Low risk	Moderate risk	Moderate quality
4	What is the safe window from definitive fixation to flap coverage in type 3B open tibia fractures? Supporting plastics and orthopaedics alliance in reducing trauma adverse events (SPARTA) [15]	Al-Hourani et al.	2023	Retrospective cohort study	N/A	High risk	Moderate risk	Moderate risk	Low risk	Low risk	Moderate-high	Low quality
5	Delay in flap coverage past 7 days increases complications for open tibia fractures: a cohort study of 140 North American trauma centers [16]	Pincus et al.	2018	Retrospective cohort study	N/A	High risk	Moderate risk	Low risk	Low risk	Low risk	Moderate risk	Moderate quality
6	The lymphatic response to injury with soft-tissue reconstruction in high-energy open tibial fractures of the lower extremity [17]	van Zanten et al.	2016	Prospective cohort study	Moderate risk	High risk	Low risk	Low risk	Moderate risk	Low risk	Moderate-high risk	Moderate quality
7	Treatment of compound tibial fracture with free osteomuscular latissimus dorsi scapula flap [18]	Junnila et al.	2015	Prospective case series	Moderate risk	High risk	High risk	Low risk	Low risk	Low risk	Moderate-high risk	Moderate quality
8	Early versus late flap coverage for open tibial fractures [19]	Chua et al.	2014	Retrospective study	N/A	High risk	Low risk	Low risk	Low risk	Low risk	Moderate risk	Moderate quality
9	Gustilo IIIB open tibial fractures: an analysis of infection and nonunion rates [20]	Singh et al.	2020	Retrospective cohort study	N/A	High risk	Moderate risk	Low risk	Low risk	Low risk	High risk	Moderate quality
10	Complications and timing of soft tissue coverage after complete articular, open tibial plateau fractures [21]	Grisdela et al.	2022	Retrospective cohort study	N/A	High risk	Moderate risk	N/A	Low risk	Low risk	High risk	Moderate quality
11	Single-stage orthoplastic reconstruction of Gustilo-Anderson Grade III open tibial fractures greatly reduces infection rates [22]	Mathews et al.	2015	Retrospective cohort study	N/A	High risk	Moderate risk	Low risk	Low risk	Moderate risk	Moderate risk	Low quality
12	Markers of blood coagulation and fibrinolysis in patients with early and delayed microsurgical reconstructions in the lower extremities [23]	Kloeters et al.	2017	Prospective cohort study	Moderate risk	High risk	Low risk	Low risk	Low risk	Low risk	Moderate risk	Moderate quality
13	Early soft tissue coverage and negative pressure wound therapy optimises patient outcomes in lower limb trauma [24]	Liu et al.	2011	Retrospective cohort study	N/A	High risk	Moderate risk	Low risk	Low risk	Low risk	Moderate risk	Moderate quality
14	When is the critical time for soft tissue reconstruction of open tibia fracture patients? [25]	Lee et al.	2021	Retrospective cohort study	N/A	High risk	Moderate risk	Low risk	Moderate risk	Low risk	Moderate risk	Moderate quality

Meta-Analysis

**Figure 2:**
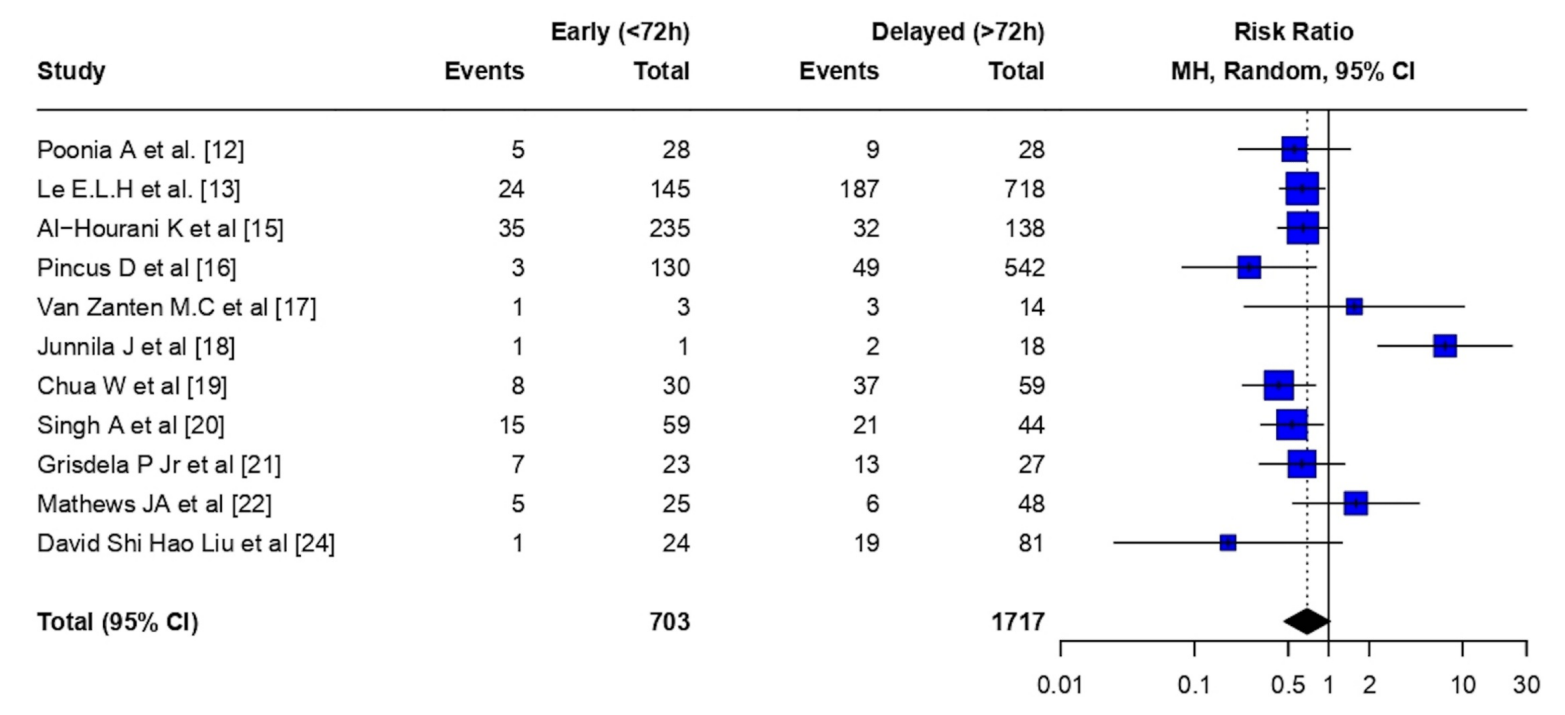
Infection - meta-analysis CI: confidence interval, MH: Mantel–Haenszel

Infections: Early flap coverage (<72 hours) was associated with a non-statistically significant reduction in infection risk compared with delayed flap coverage (>72 hours) (RR 0.69, 95% CI 0.47-1.02; p = 0.064). There was moderate-to-substantial heterogeneity among studies (τ² = 0.24; I² = 64.5% (95% CI 32.3-81.4%); H = 1.68). The Cochran Q test indicated statistically significant heterogeneity (Q = 28.14, df = 10; p = 0.0017) (Figure 2).

**Figure 3:**
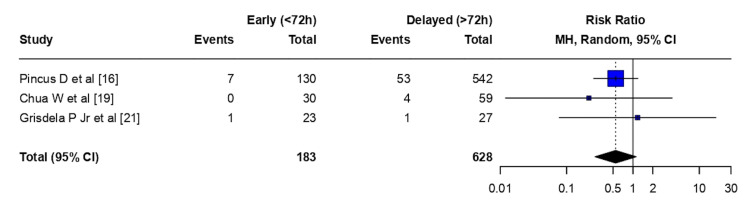
Amputation - meta-analysis CI: confidence interval, MH: Mantel–Haenszel

Amputations: Early flap coverage (<72 hours) was associated with a non-statistically significant reduction in amputation risk compared with delayed flap coverage (>72 hours) (RR 0.55, 95% CI 0.27-1.12; p = 0.099). There was no evidence of between-study heterogeneity (τ² = 0; I² = 0.0% (95% CI 0.0-89.6%); H = 1.00). The Cochran Q test did not indicate statistically significant heterogeneity among studies (Q = 0.71, df = 2; p = 0.703) (Figure 3).

**Figure 4:**
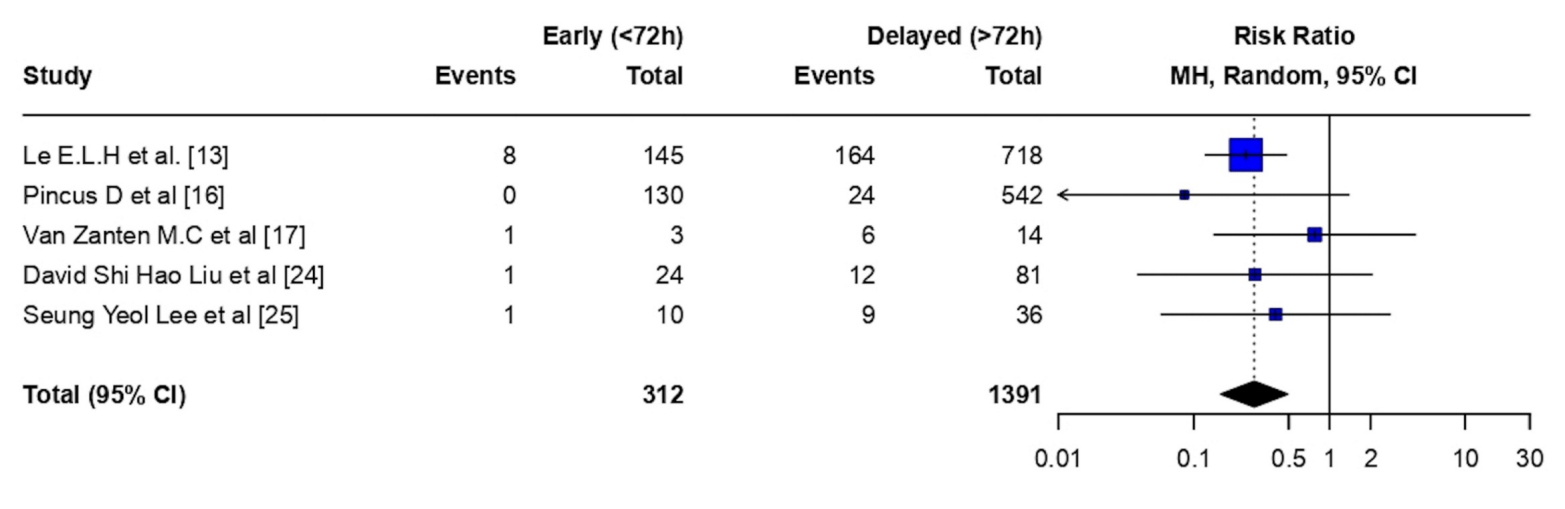
Osteomyelitis - meta-analysis CI: confidence interval, MH: Mantel–Haenszel

Osteomyelitis: Early flap coverage (<72 hours) was associated with a statistically significant reduction in the risk of osteomyelitis compared with delayed flap coverage (>72 hours) (RR 0.28, 95% CI 0.16-0.49; p < 0.0001). There was no evidence of between-study heterogeneity (τ² = 0.00; I² = 0.0% (95% CI 0.0-79.2%); H = 1.00). The Cochran Q test did not indicate statistically significant heterogeneity (Q = 2.54, df = 4; p = 0.638) (Figure 4).

**Figure 5:**
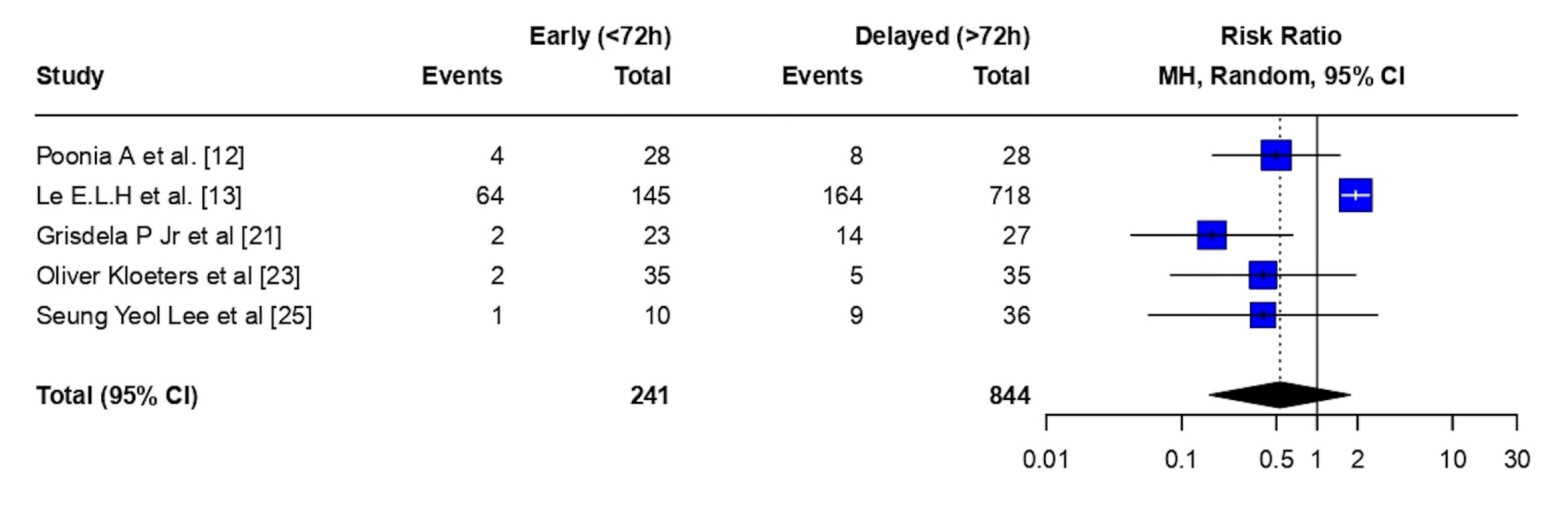
Reoperation - meta-analysis CI: confidence interval, MH: Mantel–Haenszel

Reoperation: Early flap coverage (<72 hours) was not associated with a statistically significant reduction in reoperation risk compared with delayed flap coverage (>72 hours) (RR 0.53, 95% CI 0.16-1.76; p = 0.30). There was substantial heterogeneity among studies (τ² = 1.42; I² = 84.0% (95% CI 64.1-92.9%); H = 2.50). The Cochran Q test indicated statistically significant heterogeneity (Q = 25.06, df = 4; p < 0.0001) (Figure 5).

Meta-analysis thus highlights a statistically significant reduction in the risk of osteomyelitis with early flap coverage. Infection and amputation risks had non-statistically significant reductions with early flap coverage, while reoperation risks did not show statistically significant reductions when comparing early and delayed flap coverage.

Discussion

This study demonstrates that flap coverage within 72 hours is associated with improved outcomes, including lower rates of infection, osteomyelitis, and reoperations compared with delayed reconstruction. The results highlight timing as a critical determinant of patient outcomes in complex lower-limb injuries and reaffirm current guideline recommendations advocating early reconstruction where feasible. Infection-related outcomes and reoperations showed the most notable differences, with superficial/deep infection rates rising from 14.94% to 22.02% and 30.29% to 81.75%, respectively. Meta-analyses have also demonstrated statistically significant reductions in the risk of osteomyelitis among patients undergoing early flap coverage (RR 0.28, p < 0.0001). These findings should be interpreted cautiously, given the limited number of high-quality, robust comparative studies. The overall reduction in complication incidence, however, reinforces the benefit of early soft tissue coverage following open fractures, consistent with current literature recommendations.

The oft-quoted 72-hour threshold originates from Godina et al.'s 1986 paper, which demonstrated fewer complications and shorter hospitalization with early microsurgical reconstruction [5]. In December 2017, the British Orthopedic Association introduced the British Orthopedic Association Standards for Trauma (BOAST) guideline, encouraging definitive soft tissue closure/coverage within 72 hours of injury if it cannot be performed during the time of debridement, which is recognized nationally by the National Institute for Health and Care Excellence (NICE) in the UK [26,27]. Other guidelines, such as those of the Orthopedic Trauma Association and the American College of Surgeons, follow a similar approach to early soft tissue coverage, allowing a slightly longer window of up to seven days post-injury [28]. It is hypothesized that this practice reduces bacterial colonization, enhances local vascular perfusion, and mitigates the predisposition to infection and nonunion. The studies included in this review examine the years preceding 2010, consistent with this premise and predominantly advocating flap reconstruction within 72 hours.

Clinically, the practical implications of these findings emphasize the need for early multidisciplinary coordination between prehospital services and orthoplastic teams. Prehospital services play an equally crucial role by facilitating rapid identification, early resuscitation, and prompt transfer of patients to trauma centers capable of providing limb reconstruction, which is vital to ensure flap coverage is performed within 72 hours. Pollak et al. indicated that prolonged out-of-hospital time is associated with higher infection rates. However, the prolonged period may indicate increased injury severity [29]. At a specialist center, the orthoplastic “fix and flap” approach remains a cornerstone of modern limb reconstruction, achieving prompt skeletal stabilization and definitive soft tissue coverage.

Despite this review's findings favoring early flap coverage, other factors, independent of timing, influence outcomes and warrant consideration. Patient comorbidities, diabetes, immunosuppression, smoking, and alcohol use compromise the healing potential, while warm climates and agricultural environments raise the risk of contamination and subsequent infection [30]. Furthermore, fracture severity and injury complexity determine the incidence of complications. Thakore et al. demonstrated a higher incidence of infections and nonunion in patients with Gustilo-Anderson III fractures (particularly in Gustilo-Anderson IIIC injuries) [31]. Such fractures introduce greater contamination and a higher level of soft tissue damage, compromising the vascular supply and, in turn, impairing the immune response [30]. Flap selection is another key determinant. While this review included both free and local flaps and skin grafts, several studies have investigated the outcomes for the different methods of open wound coverage. Comparative findings favor the free flap, but this comparison remains controversial; it is accepted that the choice is guided by the interplay of numerous factors [32]. Early prophylactic antibiotic administration represents another critical determinant of success. Evidence from Lack et al. indicates that infection risk is significantly reduced with prompt, adequate antibiotic use following injury. Delays beyond 66 minutes or suboptimal antibiotic selection can substantially alter the outcome of severe open fractures [33]. Finally, resource availability and the presence of orthopedic and plastic surgical facilities are more likely to achieve flap reconstruction targets within 72 hours than in resource-limited hospitals. Surgical availability, microsurgical expertise, and theater access may impose timing constraints.

This review’s strengths include its comprehensive and up-to-date search strategy, which captures the most recent comparative literature on early versus delayed flap coverage. Adherence to the PRISMA methodology provided a systematic framework for study selection, data extraction, and synthesis, reducing the risk of selection bias. Additionally, rigorous bias/quality assessments were conducted for all included studies, thereby enhancing the reliability of the pooled findings. By focusing on recent studies, this review reflects current orthoplastic practices and provides up-to-date evidence and guidance on optimal timing for flap reconstruction in open lower-limb trauma.

However, limitations should be acknowledged. First, a high degree of heterogeneity was observed in the pooled fracture flap coverage times, which limits the generalisability of these findings. This may be attributed to variability in study design, institutional protocols, resource availability, and surgical techniques used. Most studies were retrospective, with no RCTs, inconsistent outcome definitions, and varying follow-up durations. These increase the risk of selection and reporting bias and may contribute to type II error, whereby the true effect of early flap reconstruction is underestimated due to inconsistencies in data collection and analysis across studies.

Additionally, the inclusion of studies with zero-event cells required a continuity correction, which may introduce bias, particularly in studies with small sample sizes or imbalanced group sizes. It may further contribute to heterogeneity in the pooled estimates. Weighting bias may also arise from the imbalance between the smaller early-flap-coverage group (772 patients) and the larger delayed group (1882 patients), potentially affecting the precision and stability of effect estimates for the early-intervention arm. Consequently, the results should be interpreted with caution. Although our meta-analysis focused on short-term complications, it did not account for long-term clinical outcomes or patient satisfaction, which are crucial to a comprehensive evaluation of its impact.

Recent evidence indicates a continued rise in the incidence of open fractures. A trauma network audit and research network assessment by Shah et al. demonstrated that the number of open fractures in all patients has increased by almost 3x from 2008 to 2019, with lower limb fractures predominating [1]. It is therefore vital to adhere to research-based guidelines to ensure optimal treatment and patient outcomes.

Future research should prioritize prospective multicenter studies with standardized reporting of outcomes that align with national/international guidelines, capturing variables such as coverage timing, flap types, and both short- and long-term functional outcomes. Standardized reporting of complications and patient-reported outcome measures would facilitate the conduction of more robust, high-quality meta-analyses. Furthermore, the inclusion of health-economic data, such as operative/theater times, days of hospital stay, and readmission rates, would provide valuable information to ensure cost-effectiveness and quantify resource allocation.

## Conclusions

The findings within this study reinforce prior reviews and guidelines, encouraging early flap coverage (within 72 hours) due to its association with fewer complications. However, due to heterogeneity and the predominance of retrospective studies, the overall quality remains limited. Additionally, evidence indicates that timing alone does not dictate outcome, and patient-specific factors can influence the results. These findings collectively provide modern evidence to support refining existing orthoplastic guidelines toward a more evidence-based approach to flap timing in lower-limb reconstruction.
